# Adsorption and Desorption Characteristics of Cd^2+^ and Pb^2+^ by Micro and Nano-sized Biogenic CaCO_3_

**DOI:** 10.3389/fmicb.2018.00041

**Published:** 2018-01-26

**Authors:** Renlu Liu, Yong Guan, Liang Chen, Bin Lian

**Affiliations:** ^1^Jiangsu Key Laboratory for Microbes and Functional Genomics, Jiangsu Engineering and Technology Research Center for Microbiology, College of Life Sciences, Nanjing Normal University, Nanjing, China; ^2^National Synchrotron Radiation Laboratory, University of Science and Technology of China, Hefei, China

**Keywords:** biogenic CaCO_3_, heavy metals, adsorption, desorption, mechanism

## Abstract

The purpose of this study was to elucidate the characteristics and mechanisms of adsorption and desorption for heavy metals by micro and nano-sized biogenic CaCO_3_ induced by *Bacillus subtilis*, and the pH effect on adsorption was investigated. The results showed that the adsorption characteristics of Cd^2+^ and Pb^2+^ are well described by the Langmuir adsorption isothermal equation, and the maximum adsorption amounts for Cd^2+^ and Pb^2+^ were 94.340 and 416.667 mg/g, respectively. The maximum removal efficiencies were 97% for Cd^2+^, 100% for Pb^2+^, and the desorption rate was smaller than 3%. Further experiments revealed that the biogenic CaCO_3_ could maintain its high adsorption capability for heavy metals within wide pH ranges (3–8). The FTIR and XRD results showed that, after the biogenic CaCO_3_ adsorbed Cd^2+^ or Pb^2+^, it did not produce a new phase, which indicated that biogenic CaCO_3_ and heavy metal ions were governed by a physical adsorption process, and the high adsorptive capacity of biogenic CaCO_3_ for Cd^2+^ and Pb^2+^ were mainly attributed to its large total specific surface area. The findings could improve the state of knowledge about biogenic CaCO_3_ formation in the environment and its potential roles in the biogeochemical cycles of heavy metals.

## Introduction

The main current environmental problems are the increasing atmospheric greenhouse effect and environmental pollution of large areas. Moreover, the increase of population and industrial or agricultural production makes such environmental issues more prominent. Heavy metal pollution in water and soil is already a global problem, and it is especially serious in soils (Zhao et al., 2014a). For example, the proportion of heavy metal pollution has exceeded 2.5% by land area, covering 2.32 million hectares in China, and the exceedances of permissible threshold values for Cd, Hg, As, Cu, Pb, Cr, Zn, and Ni are 7, 1.6, 2.7, 2.1, 1.5, 1.1, 0.9, and 4.8%, respectively (The Ministry of Environmental Protection, 2014; Zhao et al., 2014a; The Ministry of Land and Resources, 2015).

Microbial methods are become favored due to their low cost and environmentally—friendly nature (Lian et al., 2008; Kavamura and Esposito, 2010; Moreau et al., 2013; Santos et al., 2017), but the adsorbed heavy metals may re-enter the environment and cause re-pollution with the change in environmental conditions (Pavasant et al., 2006; Pan et al., 2007; Apiratikul and Pavasant, 2008; Tsekova et al., 2010). A sequestration method seems to be the most convenient and most commonly chosen method (Lagadic et al., 2001; Babel and Kurniawan, 2003; Kobya et al., 2005; Ren et al., 2013); however, currently available heavy metals adsorbents remain limited, and most traditional adsorbents come with high utilization costs. Therefore, it is necessary to develop a new high-efficiency, low-cost, environmentally-friendly heavy metal adsorbent.

CaCO_3_ is one of the most abundant bio-minerals found in nature, and it has aroused great interest in many branches of science. The biosynthesis of CaCO_3_ is of great significance. It can promote carbon deposition, thus contributing to mitigate global warming (Dupraz et al., 2009; Mitchell et al., 2010; Phillips et al., 2013). CaCO_3_ is reported to adsorb heavy metal ions in water or soil with good effect, and increasing the amount of CaCO_3_ in the soil or water can significantly reduce the migration of heavy metals (Al-Degs et al., 2006; Yavuz et al., 2007; Aziz et al., 2008; Cai et al., 2010; Zhao et al., 2014a). However, the use of biogenic CaCO_3_ combined with microbial technology to remediate heavy metal pollution, including the related process and the microscopic mechanism, has not yet been reported.

Natural CaCO_3_ from limestone has limited further development prospects as a result of its low purity and efficiency. The traditional CO_2_-bubble method for synthesizing CaCO_3_ cannot sufficiently regulate the product size, morphology, or crystal type, and the cost is higher. But it is feasible to produce biogenic CaCO_3_ particles with morphological diversity (such as: spherical, rhabditiform, flower, dumbbell-shape, reticular structure aggregates, etc.) and low cost by microbial mineralisation technology (Lian et al., 2006; Han et al., 2013; Cao et al., 2016). On this basis, developing the application of biogenic CaCO_3_ in the treatment of heavy metals can not only deepen our understanding of the environmental significance of bio-mineralisation, but also develop a potential means with which to control heavy metal pollution.

The adsorption of heavy metal ions is subject to many factors, such as contact time, temperature, pH, and so on. Since the surface charge of an adsorbent in a solution could be altered by changing its pH value, the pH is one of the most important factors affecting the adsorption process of metal ions (Farrah and Pickering, 1979; Chen et al., 1997; Abollino et al., 2003; Üçer et al., 2006; Wolthers et al., 2008; Meng et al., 2009; Ma et al., 2012). Here, the adsorption-desorption properties of Cd^2+^ and Pb^2+^ by CaCO_3_ induced by *Bacillus subtilis* and the pH effect on adsorption were investigated. This study will improve our knowledge of biogenic CaCO_3_ formation in the environment and its potential role in the remediation of heavy metals.

## Materials and methods

### Preparation of micro and nano-sized biogenic CaCO_3_ and its morphology and chemical composition analysis

#### Experimental strain

*B. subtilis* (GenBank accession number KT343639), derived from the National Research and Extension Centre of Microbial Fertilizer Technology of China, is the legal functional microbial fertilizer strain in China.

We inoculated two or three rings with *B. subtilis* in 200 mL LB liquid culture medium [tryptone 0.1% (W/V), yeast extract 0.5% (W/V), NaCl 1% (W/V), 6.5 ≤ pH ≤ 7.5], shaking-cultured for 10 h at 30°C and 180 rpm, to prepare the bacterial liquid [(7.75 ± 1.19) × 10^7^ cfu/mL]. We added 100 mL LB liquid medium (containing CaCl_2_ 0.2 g) to a clean 250 mL conical flask. Afterwards, we inoculated 2 mL strain from the aforementioned bacterial liquid to form the experimental group, and set up 20 parallel, shaking-cultured samples (30°C, 180 rpm, for 7 days) to induce CaCO_3_ synthesis. The culture solution was centrifuged at 8000 rpm for 15 min at 4°C, and then the centrifuged sediments were collected and dried at 55°C, then milled to 200 mesh size or finer by agate mortar in readiness for testing. To verify whether the acquisition of micro- and nano-sized biogenic CaCO_3_ was successful, or not, we smeared the precipitate evenly on clean cover-glasses, drying naturally, then, subjected them to field emission scanning electron microscopy and energy dispersive spectrometry (FESEM-EDS) analysis. In addition, the XRD and TEM-SAED (selected area electron diffraction) methods were used to analyse the crystal structure of the precipitate.

### The adsorption and desorption of Cd^2+^ and Pb^2+^ by micro and nano-sized biogenic CaCO_3_

To investigate the environmental remediation benefits of biogenic CaCO_3_, the adsorption and desorption characteristics of two common heavy metal ions (Cd^2+^ and Pb^2+^) under the action of biogenic CaCO_3_ were investigated. The Langmuir and Freundlich equations were used to fit an adsorption model, and this was then employed to obtain the maximum adsorption capacity of such heavy metals (Wang et al., 2007b; Mikutta et al., 2012; Musso et al., 2014).

#### Adsorption experiments

Some 0.10 g biogenic CaCO_3_ was added to a 50 mL polyethylene centrifuge tube containing 20 mL solution with different Cd^2+^ (CdCl_2_), and Pb^2+^ (Pb(NO_3_)_2_) concentrations (0, 5, 10, 30, 60, 100, 150, 180, 220, and 260 mg/L: concentration based on actual measurements). The mixture was shaken at 25°C, and 100 rpm in a shaker for 24 h, and each group was tested as a set of three replicates. After shaking, the supernatant was separated by centrifuging at 8000 rpm for 15 min. The concentration of metal ions was determined by atomic absorption spectrometer (AAS, AA-6300C, Shimadzu). The adsorption amount of Cd^2+^ and Pb^2+^ by biogenic CaCO_3_ (*Q*_e_) was calculated based on Equation (1), the adsorption isotherms were obtained by use of *C*_e_ with *Q*_e_, and the heavy metal adsorption rates were calculated by using of Equation (2), the formulae are as follows (Argun et al., 2007; Wang et al., 2007b; Lian et al., 2008; Ma et al., 2012; Yao et al., 2013; Zhao et al., 2014b; Liu et al., 2016):

(1) $$\begin{array}{rcl} Q_{\text{e}}\left(\text{mg}/\text{g}\right) & = & \frac{C_{0}-C_{e}}{W1}\times V \end{array}$$

(2) $$\begin{array}{rcl} \text{The rate of absorption }\left(\text{\%}\right) & = & \frac{C_{0}-C_{e}}{C_{0}}\times 100 \end{array}$$

Where *C*_0_ and *C*_e_ are the initial, and equilibrium concentrations of the metal ions (mg/L), respectively; *V* represents the volume of equilibrium liquid in the centrifuge tube (L), and *W*_1_ is the mass of biogenic CaCO_3_ (g).

Experimental results were analyzed with reference to the Langmuir and Freundlich isotherms (Equations 3, 4), respectively (Grimm et al., 2008; Ma et al., 2012; Mikutta et al., 2012; Wang et al., 2015):

(3) $$\begin{array}{rcl} \text{L}:\frac{1}{Q_{\text{e}}} & = & \frac{1}{Q_{m}•K_{L}}\cdot \frac{1}{C_{\text{e}}}+\frac{1}{Q_{m}} \end{array}$$

(4) $$\begin{array}{rcl} \text{F}:\text{Log }Q_{\text{e}} & = & n_{\text{f}}\cdot \text{Log }C_{\text{e}}+\text{Log }K_{\text{f}} \end{array}$$

Where *C*_e_ denotes the equilibrium concentration of metal ions in the supernatant (mg/L), *Q*_e_ is the adsorption amount of metal ions by biogenic CaCO_3_ (mg/g), *Q*_m_ denotes the maximum adsorption amount of metal ions (mg/g), *K*_L_ is the adsorption coefficient of the Langmuir model (L/mg), *K*_f_ is the Freundlich constant, and *n*_f_ is the adsorption intensity constant of the Freundlich equation.

#### Desorption experiments

We added 20 mL desorption liquid (Dong-Mei et al., 2003; Arias et al., 2006; Gherasim and Bourceanu, 2013; 1 mol/L NaNO_3_, pH = 7.0) to the centrifugal tube with the precipitates therein after adsorbing any Cd^2+^ or Pb^2+^, then the samples were shocked at 25°C, and 100 rpm for 12 h. Afterwards, the samples were centrifuged at 8000 rpm for 15 min, and AAS was used to determine the Cd^2+^ or Pb^2+^ concentrations in supernatant (*C*_1_). Each desorption experiment was conducted in triplicate.

The desorption amount of heavy metals (*Q*_de_) (Equation 5) and the rate of desorption (Equation 6) were calculated as follows (Gao et al., 2003; Wang et al., 2007a; Zhao et al., 2014b):

(5) $$\begin{array}{rcl} Q_{\text{de}}\left(\text{mg}/\text{g}\right) & = & \frac{C_{1}\times V}{W_{1}} \end{array}$$

(6) $$\begin{array}{rcl} \text{The rate of desorption }\left(\text{\%}\right) & = & \frac{Q_{de}}{Q_{e}}\times 100 \end{array}$$

Where *Q*_de_ is the desorption amount of heavy metals (mg/g), *V* is the volume of desorption solution (L), *C*_1_ represents the metal ion concentration of desorption supernatant (mg/L), and *W*_1_ is the mass of biogenic CaCO_3_ (g).

### The mechanism of adsorption

To elucidate the adsorption mechanism of biogenic CaCO_3_, we collected the biogenic CaCO_3_ before and after adsorbing Cd^2+^ or Pb^2+^, and dried it at 55°C, Afterwards, using FTIR (NEXUS670, Thermo Nicolet), XRD (Ultima IV Multipurpose, Rigaku), FESEM-EDS, and soft X-ray microscopy techniques were used to analyse the changes in structures, morphologies and elemental compositions. Meanwhile, the adsorption and desorption of Cd^2+^ (74 mg/L) and Pb^2+^ (94 mg/L) by vaterite biogenic CaCO_3_ (prepared in LB liquid medium containing 0.8 g CaCl_2_, referenced in Section Preparation of Micro and Nano-sized

Biogenic CaCO_3_ and Its Morphology and Chemical Composition Analysis) were also studied, and the structural changes of vaterite before, and after, adsorbing Cd^2+^ or Pb^2+^ were analyzed by XRD.

### The comparison of adsorption and desorption for heavy metals by biogenic CaCO_3_ and bacterial cells

We added 100 mL LB liquid medium to a clean 250 mL conical flask, sterilized it at 115°C for 20 min, then inoculated 2 mL strain from the bacterial liquid mentioned above in the experimental group, set up 10 parallel trials, and subjected them to shaking-culturation at 30°C and 180 rpm for 7 days. Then, the culture solution was centrifuged at 8000 rpm for 15 min at 4°C, whereafter, the bacterial cells were collected and dried at 55°C, and then milled to 200 mesh or finer, by agate mortar in readiness for testing.

The biogenic CaCO_3_ including CaCO_3_ and bacterial cells was used to clarify the advantages of biogenic CaCO_3_ for heavy metals the adsorption. The adsorption and desorption experiments of Cd^2+^ (74 mg/L), and Pb^2+^ (94 mg/L) by biogenic CaCO_3_ and bacterial cells were carried out using the method described in section The Adsorption and Desorption of Cd^2+^ and Pb^2+^ by Micro and Nano-sized Biogenic CaCO_3_.

### The effect of pH on adsorption of biogenic CaCO_3_ for heavy metals

To study the influence of pH on the removal efficiency, 0.05 g biogenic CaCO_3_ was placed into a 50 mL polyethylene centrifuge tube containing 20 mL solution with different pH values (1, 2, 3, 4, 5, 6, 7, and 8) of 83.13 mg/g Cd^2+^ (CdCl_2_), or 99.30 mg/g Pb^2+^ (Pb(NO_3_)_2_), respectively. The mixture was shaken at 25°C and 100 rpm for 24 h, and each group was replicated three times. The supernatant was obtained by centrifuging at 8000 rpm for 15 min. The concentrations of Cd^2+^ or Pb^2+^ in the supernatant were determined by AAS, and the heavy metal adsorption rates were calculated by use of Equation (2).

## Results and discussion

### The morphological and elemental composition analysis of biogenic CaCO_3_ sediments

**Figure 1:**
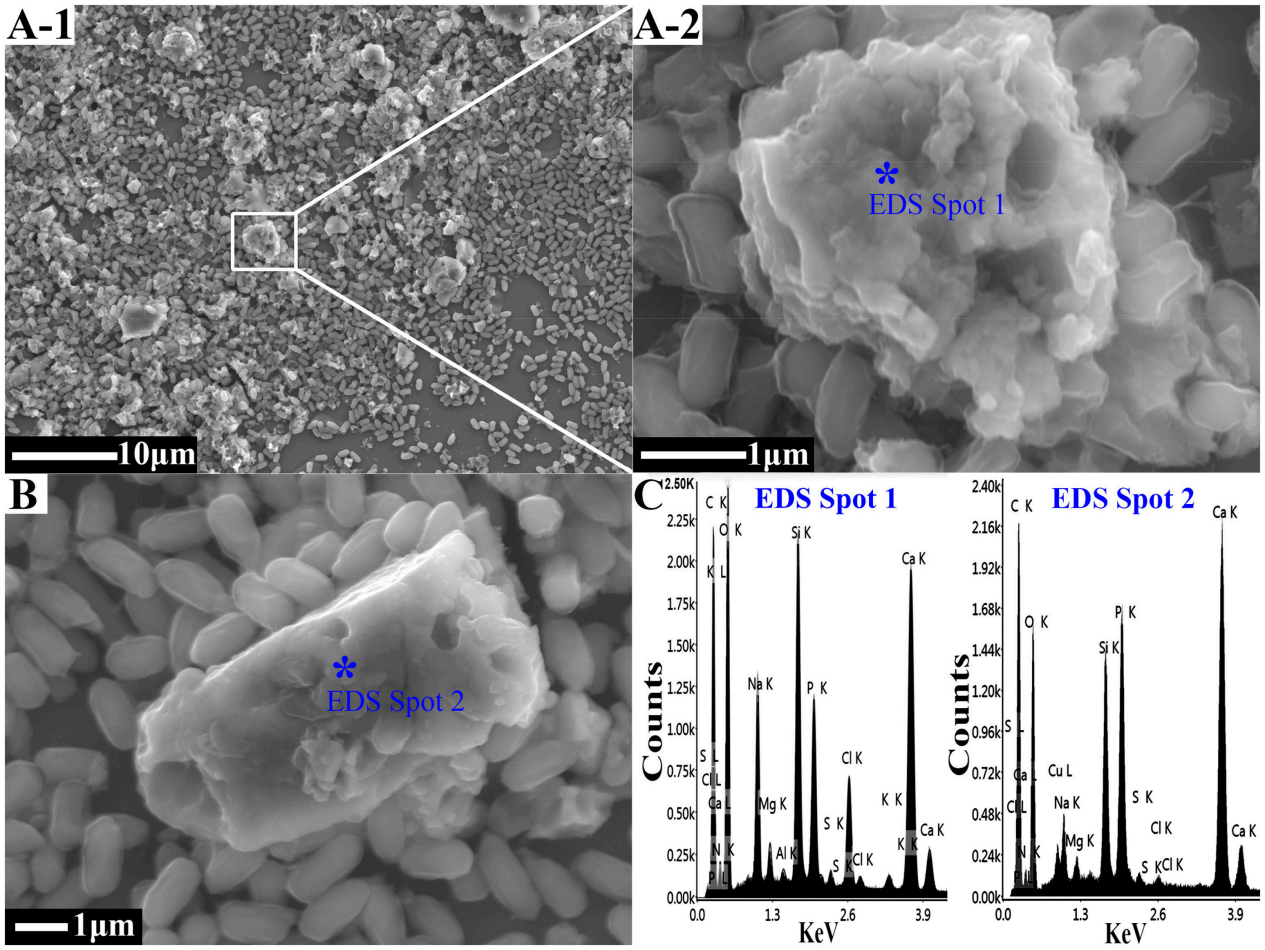
The FESEM-EDS analysis of biogenic CaCO_3_. **(A,B)** The FESEM images of the biogenic CaCO_3_; among them, **A–2** is the magnification for the marked area in **A–1**. **(C)** The EDS results for the asterisk site. The “^*^” showed the site of the EDS analysis.

Different morphologies of crystals in the sediments were observed by using FESEM. These morphologies included cauliflower-like forms, scaly aggregates, and various irregular aggregates of sediment (part of them shown in Figures 1A,B). EDS was used to determine the main component as being CaCO_3_ (Figure 1C). The biogenic CaCO_3_ exhibited its porous surface, corner-incomplete form, and visible irregular fine lines on its surface, thus it had both a larger internal and external specific surface area and pore volume.

### The adsorption and desorption of biogenic CaCO_3_ for Cd^2+^ and Pb^2+^

Although both Freundlich and Langmuir equations could be used to fit the isothermal adsorption process of Cd^2+^ and Pb^2+^ by biogenic CaCO_3_, the fitting effect of Langmuir adsorption isotherm equation was more favorable, which suggested that the adsorption process was a single-molecule adsorption process (Mikutta et al., 2012; Wang et al., 2015). The maximum adsorption amounts of Cd^2+^ and Pb^2+^ by biogenic CaCO_3_ were 94.340 and 416.667 mg/g, respectively (Table 1). CaCO_3_ is an important mineral that is ubiquitous in soils, shallow grand water aquifers and marine sediments which has good adsorption properties for heavy metals (Davis et al., 1987; Garcia-Sánchez and Alvarez-Ayuso, 2002; Al-Degs et al., 2006; Lee et al., 2007; Yavuz et al., 2007). Yavuz et al. (2007) found that the maximum adsorption capacities of Cd^2+^ and Pb^2+^ by natural CaCO_3_ were determined as 18.52 and 19.92 mg/g, respectively. This research on the adsorption of heavy metals with biogenic CaCO_3_ induced by the strain (as a legal strain used in microbial fertilizer) is the first report, and the maximum adsorption capacities of biogenic CaCO_3_ for heavy metals are apparently higher than that of natural calcite (*p* < 0.01), which suggests a considerable potential to immobilize or passivate heavy metals in contaminated soil.

**Table 1:** Isotherms coefficients according to Langmuir and Freundlich.

**Elements**	**Langmuir**			**Freundlich**		
	***K*_L_ (L/g)**	***Q*_m_ (mg/g)**	**R^2^**	**Log*K*_F_(L/g)**	**n_f_**	**R^2^**
Cd^2+^	0.033	94.340	0.994	0.531	1.056	0.981
Pb^2+^	0.004	416.667	0.953	0.255	0.976	0.914

**Figure 2:**
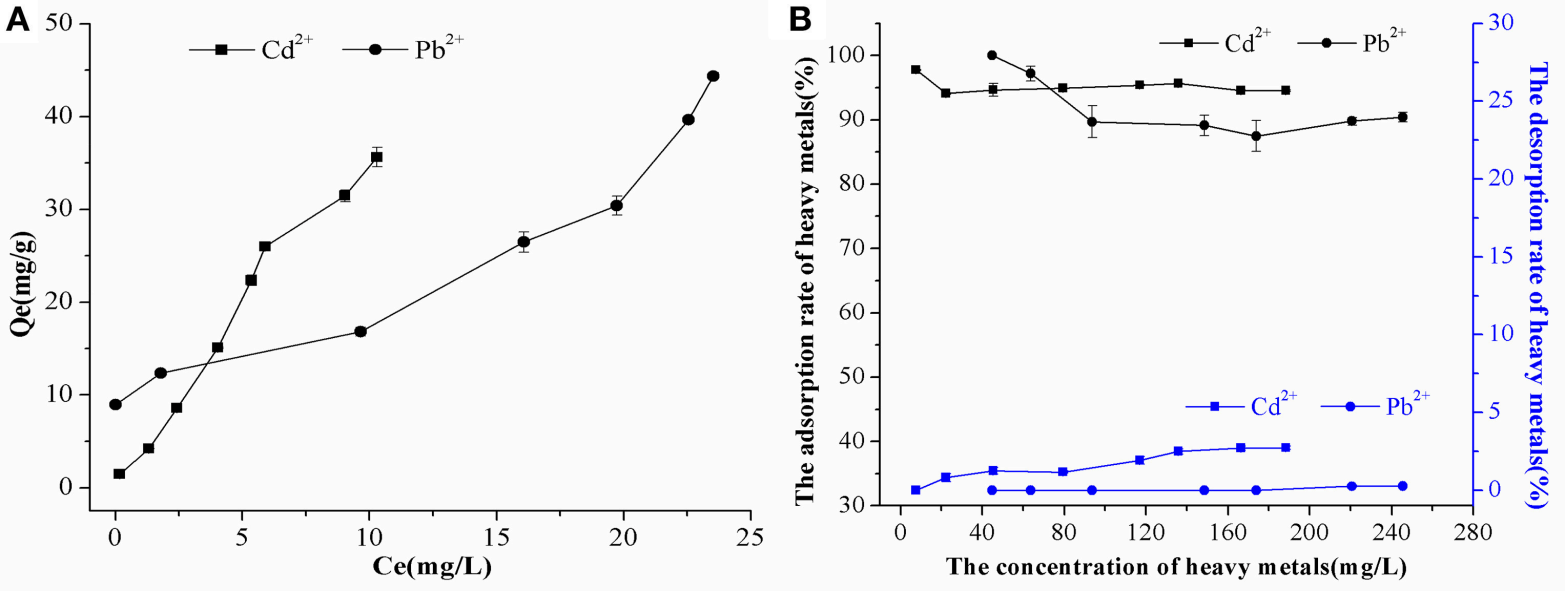
The adsorption and desorption characteristics of Cd^2+^ and Pb^2+^ by biogenic CaCO_3_. **(A)** The adsorption isotherm curves. **(B)** The adsorption and desorption rates. The black line represents the adsorption rate data, and the blue line represents the desorption rate data. Data represent the mean ± standard deviation (SD) of three independent experiments.

Figure 2A showed that the adsorption amount (*Q*_e_) of Cd^2+^ or Pb^2+^ on biogenic CaCO_3_ increased with increasing Cd^2+^ or Pb^2+^ concentration in the equilibrium solution (*C*_e_). When the concentration of Cd^2+^ or Pb^2+^ was between 5 and 260 mg/L, the rate of adsorption of heavy metals on biogenic CaCO_3_ was as high as 87–100%, while the rate of desorption remained steady at 0.1–3% (Figure 2B), which suggest that biogenic CaCO_3_ has a high adsorption capacity for heavy metals and carries little environmental risk. The results provide evidence that bacterial fertilizer and biogenic CaCO_3_ may play important roles in various environments, and indeed more than previously acknowledged.

### The adsorption mechanism

**Figure 3:**
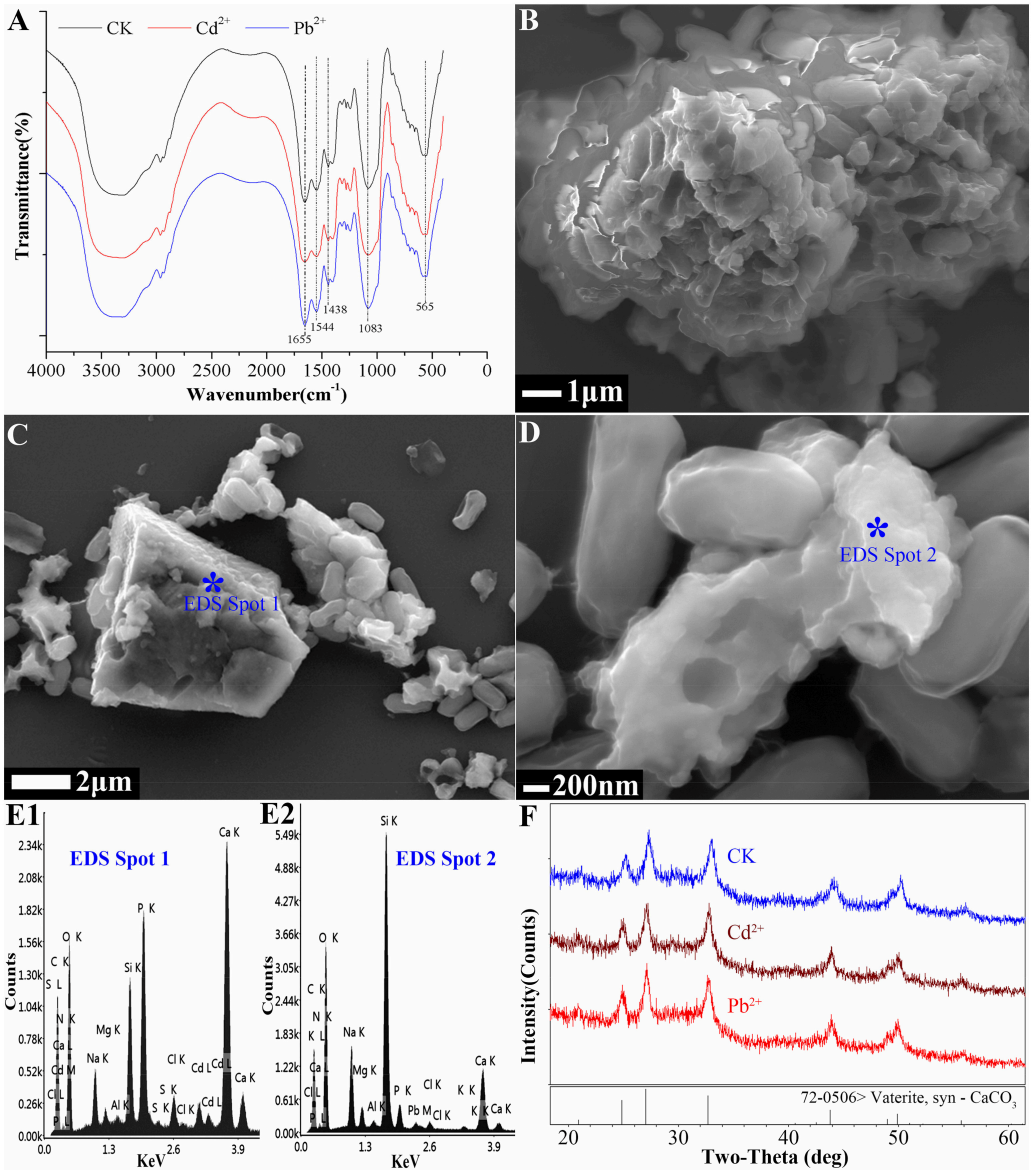
The adsorptive mechanism analysis of Cd^2+^ and Pb^2+^ by biogenic CaCO_3_. **(A)** The results of FTIR spectra of biogenic CaCO_3_ before, and after, adsorbing Cd^2+^ or Pb^2+^ (CK: before adsorbing Cd^2+^ or Pb^2+^ by biogenic CaCO_3_). **(B)** The reticular structure of biogenic CaCO_3_ by FESEM. **(C,E1)** The result of biogenic CaCO_3_ after adsorbing Cd^2+^ (74 mg/L) by FESEM-EDS: Cd^2+^ is visible on the surface of the biogenic CaCO_3_. **(D,E2)** The result of biogenic CaCO_3_ after adsorbing Pb^2+^ (94 mg/L) by FESEM-EDS: Pb^2+^ is visible on the surface of the biogenic CaCO_3_. **(F)** The XRD results of biogenic vaterite before, and after, adsorbing Cd^2+^ and Pb^2+^ (CK: before adsorbing Cd^2+^ and Pb^2+^ by biogenic CaCO_3_). The “^*^” inside the figure is the site of the EDS spot.

**Figure 4:**
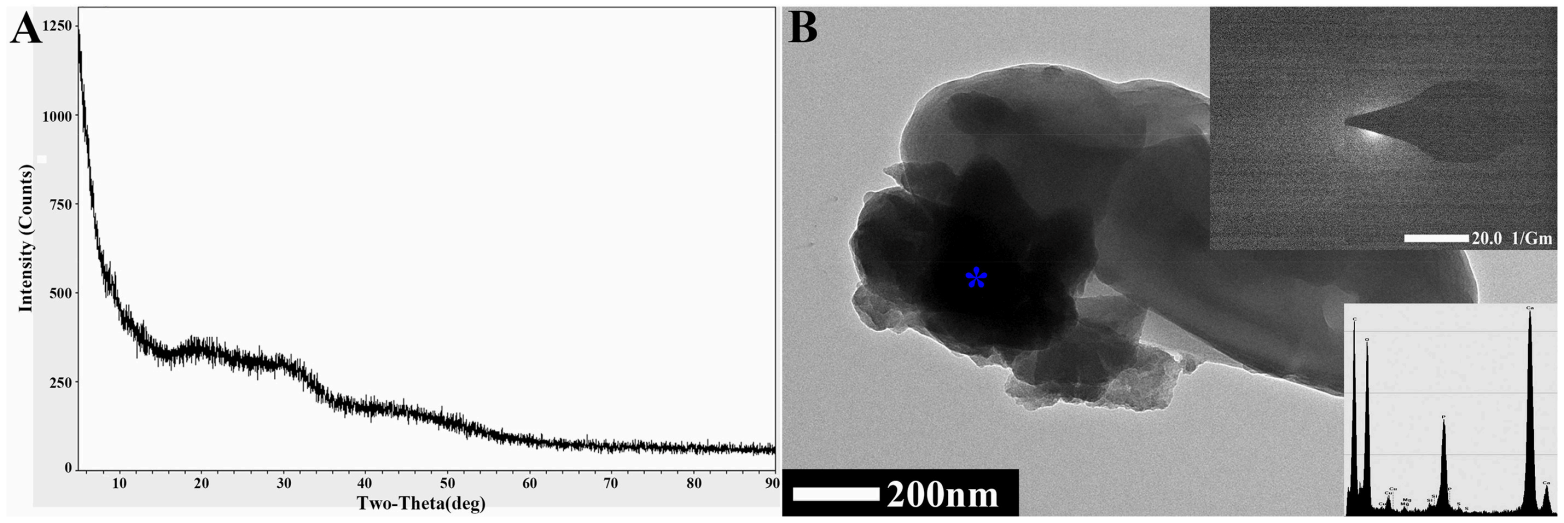
The detection and analysis of biogenic CaCO_3_ by XRD and TEM-SAED. **(A)** XRD detection. **(B)** TEM-SAED analysis. The “^*^” in the figure is the site of the EDS analysis.

The FTIR results showed that it did not undergo a chemical precipitation reaction to produce new substances after the CaCO_3_ had adsorbed Cd^2+^ (74 mg/L) or Pb^2+^ (94 mg/L) (Figure 3A); which indicated that the reaction between the biogenic CaCO_3_ and Cd^2+^ or Pb^2+^ was mainly based on physical adsorption. The FESEM-EDS analysis showed that the adsorbed Cd^2+^ and Pb^2+^ were visible on the surface of biogenic CaCO_3_ (Figures 3C,E1,D,E2). Chemical CaCO_3_ morphology is essentially a diamond-shaped cubic structure with smooth surfaces (Lian et al., 2006; Xiao et al., 2015; Cao et al., 2016), but the biogenic CaCO_3_ surface is porous, micro and nano-sized, the edge is incomplete, and it can stack to form a fragmented structure or form a reticular aggregate with other different forms of CaCO_3_ according to FESEM and soft X-ray microscopy analysis (Figures 1A,B,3B, Supplementary Video 1); thus it has a larger internal and external specific surface area and pore volume, which can provide more adsorption sites and accommodation spaces for heavy metals. The XRD and TEM-SAED results indicated that the biogenic CaCO_3_ used in this test was mainly amorphous CaCO_3_ (Figure 4), and according to the reports that the amorphous CaCO_3_ surface area is 20 times that of other crystalline forms of CaCO_3_ (Yan and Lu, 2012), therefore, it exhibits strong adsorption properties for Cd^2+^ and Pb^2+^.

Since there were no diffraction peaks observed from the amorphous biogenic CaCO_3_ in the XRD result (Figure 4A), to clarify the adsorptive mechanism of biogenic CaCO_3_ for heavy metals, vaterite-type biogenic CaCO_3_ was used to adsorb Cd^2+^ (74 mg/L) or Pb^2+^ (94 mg/L), and the adsorption rates were 98.42 and 100%, respectively, moreover, the desorption rates were all zero. The XRD results revealed that, there was no new phase diffraction peak after the biogenic vaterite had adsorbed the Cd^2+^ and Pb^2+^ (Figure 3F), which also showed that the reaction between the biogenic CaCO_3_ and Cd^2+^ or Pb^2+^ was mainly based on physical adsorption. It was also indicated that the Cd^2+^ or Pb^2+^ was stable in the mineral as a result of binding to the CaCO_3_ surface adsorption sites, or entry to the CaCO_3_ crystal pores. Consequently, biogenic CaCO_3_ offered better adsorption properties for heavy metals. Our findings suggested that the biogenic CaCO_3_ could be expected to be developed into a new type of heavy metals adsorbent, and might achieve the dual environmental benefits of carbon sequestration and heavy metal immobilization.

### The comparison of adsorption and desorption of Cd^2+^ and Pb^2+^ by biogenic CaCO_3_ and bacterial cells

**Figure 5:**
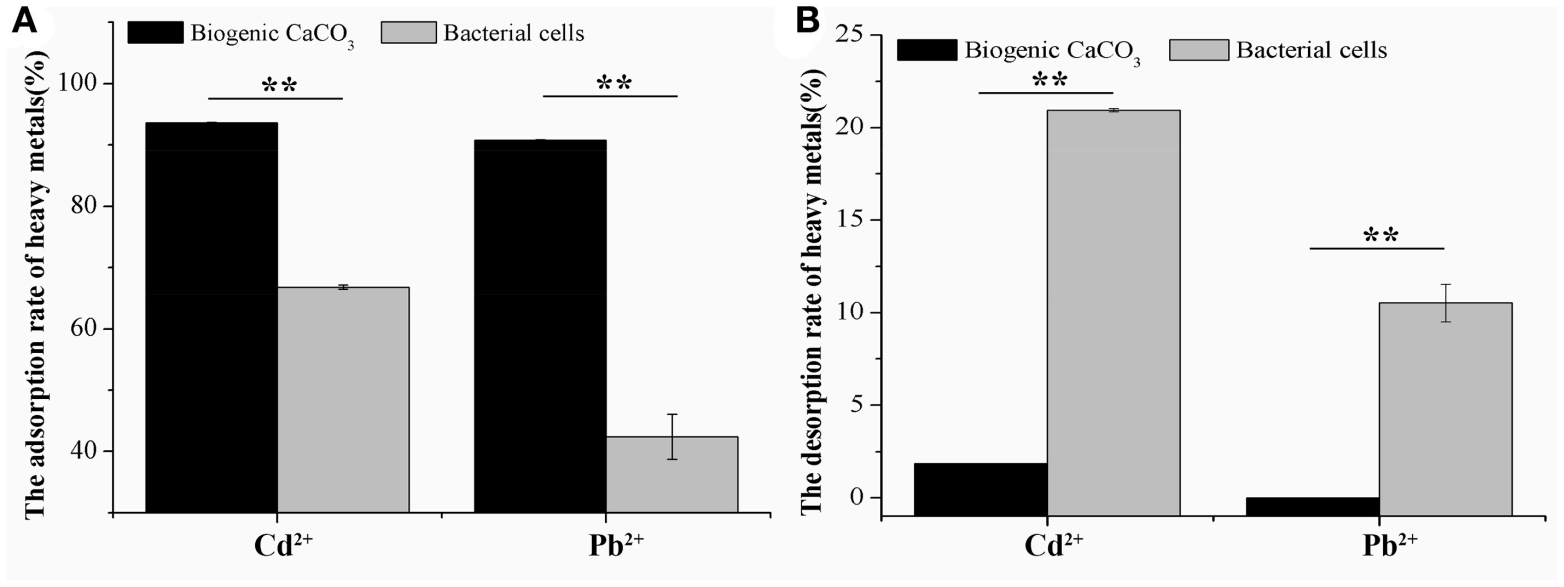
The adsorption and desorption of Cd^2+^ or Pb^2+^ by biogenic CaCO_3_ and bacterial cells. **(A)** The rate of adsorption (%). **(B)** The rate of desorption (%). The “^*^” indicates statistically significant differences between the two treatments (*t*-test, ^*^p < 0.05, ^**^p < 0.01). Data represent the mean ± SD of three independent experiments.

Figure 5 illustrates that the adsorption rate of Cd^2+^ (74 mg/L) and Pb^2+^ (94 mg/L) by biogenic CaCO_3_, was significantly higher than that of the bacterial cells, and the desorption rate was significantly smaller than that of the desorption rate of bacterial cells (*p* < 0.01), it suggested that the adsorption of CaCO_3_ crystal for heavy metals was dominant and its environmental risk was very low, but the adsorption rate of bacterial cells for heavy metals was not only low, but also the adsorbed heavy metals would be released to the environment easily, therefore, it posed a higher environmental risk. This also suggested that the biogenic CaCO_3_ had a larger specific surface area and rich reticular structures which contributed to its high adsorption and low desorption performance. This significant retaining ability of heavy metal ions indicates the remarkable efficiency of biogenic CaCO_3_ as an adsorbent.

### The effect of pH on adsorption of biogenic CaCO_3_ for heavy metals

**Figure 6:**
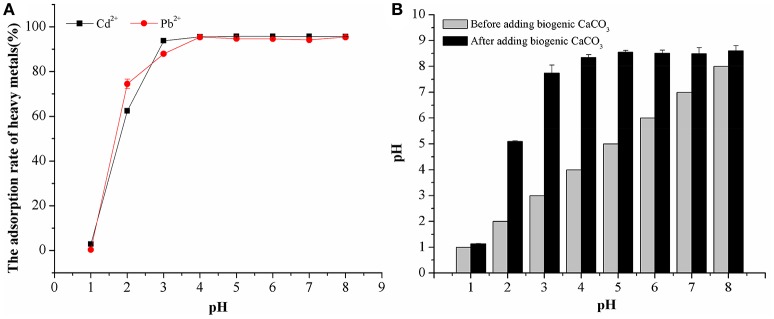
The effect of pH on the adsorption of heavy metals by biogenetic CaCO_3_**. (A)** The adsorption rate of biogenetic CaCO_3_ for heavy metals under different pH conditions (%). **(B)** The variation of pH before, and after, adding biogenetic CaCO_3_. Data represent the mean ±SD of three independent experiments.

The results in Figure 6A demonstrate the effects of pH on Cd^2+^ and Pb^2+^ adsorption by biogenic CaCO_3_. The adsorption rate of these heavy metals was quite low at pH ≤ 1, as biogenic CaCO_3_ could not exist at such a low pH value. At pH values from 1.0 to 4.0, the adsorption percentage increased rapidly with increasing pH; thereafter (pH >4) it did not change to any significant extent with further increases in pH and the adsorption percentage was stable at around 95% (Figure 6A). Similar experimental results, such as those from Ma et al. (2012) who used use chemogenic CaCO_3_ for the adsorption of Cd^2+^ and Pb^2+^ and Merrikhpour and Jalali (2012) who used natural CaCO_3_ for Cd^2+^, Pb^2+^, Cu^2+^, Zn^2+^ adsorption, etc., can also obtain good adsorption effect when starting from an acidic pH value. Furthermore, we found that the pH value of the adsorption system was increased after adding biogenic CaCO_3_, and the final pH value after adsorption is around 8.61 (Figure 6B), which should be attributable by the biogenic CaCO_3_ and alkaline metabolites produced by *B. subtilis*. In addition, the adsorption percentages of Cd^2+^ and Pb^2+^ at pH 8 were 16.22 and 41.23% when we did not add biogenic CaCO_3_, which were significantly lower than biogenic CaCO_3_ adsorption percentages (*p* < 0.01). This indicated that the adsorption rate of heavy metals was mainly influenced by the biogenic CaCO_3_ rather than the formation of heavy metal hydroxides in alkaline conditions. In summary, the high adsorption capability of the biogenic CaCO_3_ within a wide pH range (3–8) indicated its potential application in the control of the fate of heavy metals in the natural environment.

## Conclusions

The Langmuir isotherm was preferred to describe the adsorption characteristics of Cd^2+^ and Pb^2+^ by biogenic CaCO_3_ which suggested that the adsorption process was a single molecule layer adsorption process, and the maximum adsorption amounts (*Q*_m_) of Cd^2+^ and Pb^2+^ were 94.340 and 416.667 mg/g, respectively. Moreover, biogenic CaCO_3_ could maintain a high adsorption capability for heavy metals within a wide pH range. The biogenic CaCO_3_ and heavy metal ions formed a physical adsorption process, and the high efficiency and stability of the adsorption of biogenic CaCO_3_ for Cd^2+^ and Pb^2+^ were mainly attributed to its large total specific surface area and their porous structure. These findings revealed a new perspective on the remediation of heavy metal pollution by using biogenic CaCO_3_.

## Author contributions

BL: designed this study; RL, YG, and LC: performed the laboratory work; RL, YG, LC, and BL: analyzed the data; RL and BL: wrote this manuscript; All authors have read and approved the final manuscript.

### Conflict of interest statement

The authors declare that the research was conducted in the absence of any commercial or financial relationships that could be construed as a potential conflict of interest.

## Supplementary material

Supplementary Video 1 The video of biogenic CaCO_3_ by soft x-ray microscopy.

## References

[B1] Abollino O. Aceto M. Malandrino M. Sarzanini C. Mentasti E. (2003). Adsorption of heavy metals on Na-montmorillonite. Effect of pH and organic substances. Water Res. 37, 1619–1627. 10.1016/S0043-1354(02)00524-9 12600390

[B2] Al-Degs Y. S. El-Barghouthi M. I. Issa A. A. Khraisheh M. A. Walker G. M. (2006). Sorption of Zn (II), Pb (II), and Co (II) using natural sorbents: equilibrium and kinetic studies. Water Res. 40, 2645–2658. 10.1016/j.watres.2006.05.018 16839582

[B3] Apiratikul R. Pavasant P. (2008). Batch and column studies of biosorption of heavy metals by *Caulerpa lentillifera*. Bioresour. Technol. 99, 2766–2777. 10.1016/j.biortech.2007.06.036 17698354

[B4] Argun M. E. Dursun S. Ozdemir C. Karatas M. (2007). Heavy metal adsorption by modified oak sawdust: thermodynamics and kinetics. J. Hazard. Mater. 141, 77–85. 10.1016/j.jhazmat.2006.06.095 16879919

[B5] Arias M. Pérez-Novo C. López E. Soto B. (2006). Competitive adsorption and desorption of copper and zinc in acid soils. Geoderma 133, 151–159. 10.1016/j.geoderma.2005.07.002 15927557

[B6] Aziz H. A. Adlan M. N. Ariffin K. S. (2008). Heavy metals (Cd, Pb, Zn, Ni, Cu and Cr(III)) removal from water in Malaysia: post treatment by high quality limestone. Bioresour. Technol. 99, 1578–1583. 10.1016/j.biortech.2007.04.007 17540556

[B7] Babel S. Kurniawan T. A. (2003). Low-cost adsorbents for heavy metals uptake from contaminated water: a review. J. Hazard. Mater. 97, 219–243. 10.1016/S0304-3894(02)00263-7 12573840

[B8] Cai G. B. Zhao G. X. Wang X. K. Yu S. H. (2010). Synthesis of polyacrylic acid stabilized amorphous calcium carbonate nanoparticles and their application for removal of toxic heavy metal ions in water. J. Phys. Chem C 114, 12948–12954. 10.1021/jp103464p

[B9] Cao C. Jiang J. Sun H. Huang Y. Tao F. Lian B. (2016). Carbonate mineral formation under the influence of limestone-colonizing actinobacteria: morphology and polymorphism. Front. Microbiol. 7:366. 10.3389/fmicb.2016.00366 27148166 PMC4834437

[B10] Chen X. B. Judith V. W. Conca J. L. Peurrung L. M. (1997). Effects of pH on heavy metal sorption on mineral apatite. Environ. Sci. Technol. 31, 624–631. 10.1021/es950882f

[B11] Davis J. A. Fuller C. C. Cook A. D. (1987). A model for trace metal sorption processes at the calcite surface: adsorption of Cd^2+^ and subsequent solid solution formation. Geochim. Cosmochim. Acta 51, 1477–1490. 10.1016/0016-7037(87)90330-9

[B12] Dong-Mei Z. Huai-Man C. Shen-Qiang W. Chun-Rong Z. (2003). Effects of organic acids, o-phenylenediamine and pyrocatechol on cadmium adsorption and desorption in soil. Water Air Soil Pollut. 145, 109–121. 10.1023/A:1023636330221

[B13] Dupraz C. Reid R. P. Braissant O. Decho A. W. Norman R. S. Visscher P. T. (2009). Processes of carbonate precipitation in modern microbial mats. Earth Sci. Rev. 96, 141–162. 10.1016/j.earscirev.2008.10.005

[B14] Farrah H. Pickering W. F. (1979). pH effects in the adsorption of heavy metal ions by clays. Chem. Geol. 25, 317–326. 10.1016/0009-2541(79)90063-9

[B15] Gao Y. Kan A. T. Tomson M. B. (2003). Critical evaluation of desorption phenomena of heavy metals from natural sediments. Environ. Sci. Technol. 37, 5566–5573. 10.1021/es034392w 14717165

[B16] Garcia-Sánchez A. Alvarez-Ayuso E. (2002). Sorption of Zn, Cd and Cr on calcite. Application to purification of industrial wastewaters. Miner. Eng. 15, 539–547. 10.1016/S0892-6875(02)00072-9

[B17] Gherasim C. Bourceanu G. (2013). Removal of chromium (VI) from aqueous solutions using a polyvinyl-chloride inclusion membrane: experimental study and modelling. Chem. Eng. J. 220, 24–34. 10.1016/j.cej.2013.01.058

[B18] Grimm A. Zanzi R. Björnbom E. Cukierman A. L. (2008). Comparison of different types of biomasses for copper biosorption. Bioresour. Technol. 99, 2559–2565. 10.1016/j.biortech.2007.04.036 17570656

[B19] Han J. Lian B. Ling H. (2013). Induction of calcium carbonate by *Bacillus cereus*. Geomicrobiol. J. 30, 682–689. 10.1080/01490451.2012.758194

[B20] Kavamura V. N. Esposito E. (2010). Biotechnological strategies applied to the decontamination of soils polluted with heavy metals. Biotechnol. Adv. 28, 61–69. 10.1016/j.biotechadv.2009.09.002 19778598

[B21] Kobya M. Demirbas E. Senturk E. Ince M. (2005). Adsorption of heavy metal ions from aqueous solutions by activated carbon prepared from apricot stone. Bioresour. Technol. 96, 1518–1521. 10.1016/j.biortech.2004.12.005 15939281

[B22] Lagadic I. L. Mitchell M. K. Payne B. D. (2001). Highly effective adsorption of heavy metal ions by a thiol-functionalized magnesium phyllosilicate clay. Environ. Sci. Technol. 35, 984–990. 10.1021/es001526m 11351546

[B23] Lee M. Paik I. S. Kim I. Kang H. Lee S. (2007). Remediation of heavy metal contaminated groundwater originated from abandoned mine using lime and calcium carbonate. J. Hazard. Mater. 144, 208–214. 10.1016/j.jhazmat.2006.10.007 17101213

[B24] Lian B. Chen Y. Zhao J. Teng H. H. Zhu L. Yuan S. (2008). Microbial flocculation by *Bacillus* mucilaginosus: applications and mechanisms. Bioresour. Technol. 99, 4825–4831. 10.1016/j.biortech.2007.09.045 17967531

[B25] Lian B. Hu Q. Chen J. Ji J. Teng H. H. (2006). Carbonate biomineralization induced by soil bacterium *Bacillus megaterium*. Geochim. Cosmochim. Acta 70, 5522–5535. 10.1016/j.gca.2006.08.044

[B26] Liu J. Ge X. Ye X. Wang G. Zhang H. Zhou H. (2016). 3D graphene/δ-MnO 2 aerogels for highly efficient and reversible removal of heavy metal ions. J. Mater. Chem. A 4, 1970–1979. 10.1039/C5TA08106H

[B27] Ma X. Li L. Yang L. Su C. Wang K. Yuan S. (2012). Adsorption of heavy metal ions using hierarchical CaCO_3_-maltose meso/macroporous hybrid materials: adsorption isotherms and kinetic studies. J. Hazard. Mater. 209–210, 467 10.1016/j.jhazmat.2012.01.054 22326246

[B28] Meng Y. T. Zheng Y. M. Zhang L. M. He J. Z. (2009). Biogenic Mn oxides for effective adsorption of Cd from aquatic environment. Environ. Pollut. 157, 2577–2583. 10.1016/j.envpol.2009.02.035 19345460

[B29] Merrikhpour H. Jalali M. (2012). Waste calcite sludge as an adsorbent for the removal of cadmium, copper, lead, and zinc from aqueous solutions. Clean Technol. Environ. 14, 845–855. 10.1007/s10098-012-0450-0

[B30] Mikutta R. Baumgärtner A. Schippers A. Haumaier L. Guggenberger G. (2012). Extracellular polymeric substances from *Bacillus subtilis* associated with minerals modify the extent and rate of heavy metal sorption. Environ. Sci. Technol. 46, 3866–3873. 10.1021/es204471x 22443088

[B31] Mitchell A. C. Dideriksen K. Spangler L. H. Cunningham A. B. Gerlach R. (2010). Microbially enhanced carbon capture and storage by mineral-trapping and solubility-trapping. Environ. Sci. Technol. 44, 5270–5276. 10.1021/es903270w 20540571

[B32] Moreau J. W. Fournelle J. H. Banfield J. F. (2013). Quantifying heavy metals sequestration by sulfate-reducing bacteria in an Acid mine drainage-contaminated natural wetland. Front. Microbiol. 4:43. 10.3389/fmicb.2013.00043 23487496 PMC3594707

[B33] Musso T. B. Parolo M. E. Pettinari G. Francisca F. M. (2014). Cu (II) and Zn (II) adsorption capacity of three different clay liner materials. J. Environ. Manage. 146, 50–58. 10.1016/j.jenvman.2014.07.026 25156265

[B34] Pan J. Liu R. Tang H. (2007). Surface reaction of *Bacillus cereus* biomass and its biosorption for lead and copper ions. J. Environ. Sci. China 19, 403–408. 10.1016/S1001-0742(07)60067-9 17915701

[B35] Pavasant P. Apiratikul R. Sungkhum V. Suthiparinyanont P. Wattanachira S. Marhaba T. F. (2006). Biosorption of Cu^2+^, Cd^2+^, Pb^2+^, and Zn^2+^ using dried marine green macroalga *Caulerpa lentillifera*. Bioresour. Technol. 97, 2321–2329. 10.1016/j.biortech.2005.10.032 16330209

[B36] Phillips A. J. Lauchnor E. Eldring J. Esposito R. Mitchell A. C. Gerlach R.. (2013). Potential CO_2_ leakage reduction through biofilm-induced calcium carbonate precipitation. Environ. Sci. Technol. 47, 142–149. 10.1021/es301294q 22913538

[B37] Ren Y. Abbood H. A. He F. Peng H. Huang K. (2013). Magnetic EDTA-modified chitosan/SiO_2_/Fe_3_O_4_ adsorbent: preparation, characterization, and application in heavy metal adsorption. Chem. Eng. J. 226, 300–311. 10.1016/j.cej.2013.04.059

[B38] Santos D. K. Resende A. H. de Almeida D. G. (2017). Candida lipolytica UCP0988 biosurfactant: potential as a bioremediation agent and in formulating a commercial related product. Front. Microbiol. 8:767. 10.3389/fmicb.2017.00767 28507538 PMC5410559

[B39] The Ministry of Environmental Protection (2014). The Ministry of Land and Resources Report on the National Soil Contamination Survey. Available online at: http://www.mep.gov.cn/gkml/hbb/qt/201404/t20140417_270670.htm (Accessed April 17, 2014).

[B40] The Ministry of Land Resources (2015). Geological Survey Bureau of China, The Report on the Geochemical Survey of Cultivated Land in China. Available online at: http://www.cgs.gov.cn/xwl/ddyw/201603/t20160309_302254.html (Accessed June 25, 2015).

[B41] Tsekova K. Todorova D. Dencheva V. Ganeva S. (2010). Biosorption of copper(II) and cadmium(II) from aqueous solutions by free and immobilized biomass of *Aspergillus niger*. Bioresour. Technol. 101, 1727–1731. 10.1016/j.biortech.2009.10.012 19906526

[B42] Üçer A. Uyanik A. Aygün S. F. (2006). Adsorption of Cu (II), Cd (II), Zn (II), Mn (II) and Fe (III) ions by tannic acid immobilised activated carbon. Sep. Purif. Technol. 47, 113–118. 10.1016/j.seppur.2005.06.012

[B43] Wang B. Lehmann J. Hanley K. Hestrin R. Enders A. (2015). Adsorption and desorption of ammonium by maple wood biochar as a function of oxidation and pH. Chemosphere 138, 120–126. 10.1016/j.chemosphere.2015.05.062 26057391

[B44] Wang L. K. Hung Y.-T. Shammas N. K. (2007a). Advanced Physicochemical Treatment Technologies. Totowa, NJ: Humana Press.

[B45] Wang S. Nan Z. Zeng J. Hu T. (2007b). Desorption of zinc by the kaolin from Suzhou, China. Appl. Clay Sci. 37, 221–225. 10.1016/j.clay.2006.12.003

[B46] Wolthers M. Charlet L. Van Cappellen P. (2008). The surface chemistry of divalent metal carbonate minerals; a critical assessment of surface charge and potential data using the charge distribution multi-site ion complexation model. Am. J. Sci. 308, 905–941. 10.2475/08.2008.02

[B47] Xiao L. Lian B. Hao J. Liu C. Wang S. (2015). Effect of carbonic anhydrase on silicate weathering and carbonate formation at present day CO_2_ concentrations compared to primordial values. Sci. Rep. 5:7733 10.1038/srep07733 25583135 PMC4291579

[B48] Yan X. Lu Y. (2012). The Key Technologies of Light and Nano Calcium Carbonate. Beijing: Chemical Industry Press.

[B49] Yao M. Lian B. Dong H. Hao J. Liu C. (2013). Iron and lead ion adsorption by microbial flocculants in synthetic wastewater and their related carbonate formation. J. Environ. Sci. China 25, 2422–2428. 10.1016/S1001-0742(12)60151-X 24649673

[B50] Yavuz Ö. Guzel R. Aydin F. Tegin I. Ziyadanogullari R. (2007). Removal of cadmium and lead from aqueous solution by calcite. Pol. J. Environ. Stud. 16, 467–471. Available online at: https://www.researchgate.net/publication/279574155

[B51] Zhao F. Ma Y. Zhu Y. Tang Z. McGrath S. P. (2014a). Soil contamination in China: current status and mitigation strategies. Environ. Sci. Technol. 49, 750–759. 10.1021/es5047099 25514502

[B52] Zhao X. Jiang T. Du B. (2014b). Effect of organic matter and calcium carbonate on behaviors of cadmium adsorption–desorption on/from purple paddy soils. Chemosphere 99, 41–48. 10.1016/j.chemosphere.2013.09.030 24289979

